# Estimating the Number of Persons Who Inject Drugs in the United States by Meta-Analysis to Calculate National Rates of HIV and Hepatitis C Virus Infections

**DOI:** 10.1371/journal.pone.0097596

**Published:** 2014-05-19

**Authors:** Amy Lansky, Teresa Finlayson, Christopher Johnson, Deborah Holtzman, Cyprian Wejnert, Andrew Mitsch, Deborah Gust, Robert Chen, Yuko Mizuno, Nicole Crepaz

**Affiliations:** 1 Division of HIV/AIDS Prevention, National Center for HIV/AIDS, Viral Hepatitis, STD, and TB Prevention, Centers for Disease Control and Prevention, Atlanta, Georgia, United States of America; 2 Division of Viral Hepatitis, National Center for HIV/AIDS, Viral Hepatitis, STD, and TB Prevention, Centers for Disease Control and Prevention, Atlanta, Georgia, United States of America; Johns Hopkins School of Public Health, United States of America

## Abstract

**Background:**

Injection drug use provides an efficient mechanism for transmitting bloodborne viruses, including human immunodeficiency virus (HIV) and hepatitis C virus (HCV). Effective targeting of resources for prevention of HIV and HCV infection among persons who inject drugs (PWID) is based on knowledge of the population size and disparity in disease burden among PWID. This study estimated the number of PWID in the United States to calculate rates of HIV and HCV infection.

**Methods:**

We conducted meta-analysis using data from 4 national probability surveys that measured lifetime (3 surveys) or past-year (3 surveys) injection drug use to estimate the proportion of the United States population that has injected drugs. We then applied these proportions to census data to produce population size estimates. To estimate the disease burden among PWID by calculating rates of disease we used lifetime population size estimates of PWID as denominators and estimates of HIV and HCV infection from national HIV surveillance and survey data, respectively, as numerators. We calculated rates of HIV among PWID by gender-, age-, and race/ethnicity.

**Results:**

Lifetime PWID comprised 2.6% (95% confidence interval: 1.8%–3.3%) of the U.S. population aged 13 years or older, representing approximately 6,612,488 PWID (range: 4,583,188–8,641,788) in 2011. The population estimate of past-year PWID was 0.30% (95% confidence interval: 0.19 %–0.41%) or 774,434 PWID (range: 494,605–1,054,263). Among lifetime PWID, the 2011 HIV diagnosis rate was 55 per 100,000 PWID; the rate of persons living with a diagnosis of HIV infection in 2010 was 2,147 per 100,000 PWID; and the 2011 HCV infection rate was 43,126 per 100,000 PWID.

**Conclusion:**

Estimates of the number of PWID and disease rates among PWID are important for program planning and addressing health inequities.

## Introduction

Injection drug use provides an efficient mechanism for transmitting bloodborne viruses, including human immunodeficiency virus (HIV), hepatitis C virus (HCV), and hepatitis B virus (HBV). In the United States (U.S.), 8% of all new HIV infections in 2010 were among persons who inject drugs (PWID) and 3% were among PWID who also engaged in male-male sex [1]. In 2010, PWID comprised 22% of adults and adolescents living with HIV infection in the United States [2]. PWID are estimated to comprise about 16% of persons with acute HBV infection [3]. A national probability survey, conducted from 1999 through 2002, showed that 48% of adults aged 20–59 years who tested antibody positive for HCV reported a history of injection drug use [4].

The disparity in disease burden among PWID compared to their population size has been difficult to quantify. Although the Centers for Disease Control and Prevention (CDC) routinely uses population data from the Census Bureau to calculate disease rates by selected demographic categories (e.g., sex, race/ethnicity, and age at diagnosis) [3], [5] no census data are available for the number of PWID in the U.S. and rate calculations require this number for the denominator. Rates allow for comparison among subgroups and over time. Several methods have been used by various countries to measure the size of populations of PWID, including: 1) the capture-recapture method, using data collected from the population at risk; 2) the multiplier method, based on existing data; and 3) the network scale-up method, based on data collected from the general population [6]. For the U.S., multiple data sources have been compiled to estimate the population size of PWID among the nation as a whole and for large metropolitan areas [7]–[10]. While these estimates are informative, they are based on past-year behavior, which is not the most relevant time period for calculating disease rates from national HIV surveillance data, which essentially measures lifetime behaviors [5], or for calculating rates from national hepatitis C survey data since ever use of injection drugs, even in the distant past, is a risk for HCV infection [11].

Recently, CDC used meta-analysis to estimate the proportion of the U.S. population who are men who have sex with men (MSM) and quantify the burden of HIV and sexually transmitted diseases among MSM [12]. Population size estimates together with census and surveillance data were used to calculate disease rates among MSM. Applying this established method, in this report we estimate the population proportion of PWID and quantify the burden of HIV and HCV infections among PWID. We conducted a meta-analysis of national surveys to estimate the proportion of persons in the U.S. who have injected drugs, used these estimates to calculate disease metrics for PWID using national surveillance data for HIV infection, and calculated rate ratios by gender, race/ethnicity, and age. We used other methods to estimate rates of HIV infection among PWID in Puerto Rico and rates of HCV infection among adult PWID in the U.S. The estimates of the number of PWID and rates of HIV and HCV infection among PWID are needed to effectively plan, implement at an appropriate scale, and evaluate programs that serve PWID with or at risk for bloodborne infections such as HIV, HBV, or HCV.

## Methods

### Data Sources for Calculating the Proportion of PWID in the U.S

To identify data sources and methods for estimating the population proportion of PWID in the United States, we developed search strategies to identify relevant reports published from 1993 through 2008 (see Checklist S1). The initial phase (Phase 1) included an automated, systematic search of 5 electronic databases (Medline, CINAHL, PsycInfo, Sociological Abstracts, and Cochrane). We cross-referenced multiple search terms (i.e., keywords and each database's index terms) in 3 domains: (1) measurement descriptors (prevalence, epidemiologic methods, measure, survey, assessment); (2) injection drug use (substance/drug, use/abuse, intravenous/inject/parenteral); and (3) geography (some or all of the U.S.). We kept search terms broad to avoid overlooking any relevant references that might provide data or different methods for estimating the PWID population proportion.

Two trained reviewers screened abstracts from each of the 2,695 unduplicated references for specific criteria. To avoid over-estimation of population size, we excluded studies likely to have disproportionately high proportions of PWID (e.g., studies among HIV or HCV infected persons; studies focused on populations who are mentally ill, incarcerated, or in drug treatment; and studies that selected for PWID and non-PWID separately). We also excluded school-based samples (including university students) as these may under-estimate the PWID population proportion. We included studies if they reported the proportion of PWID in the sample. We screened conference abstracts and dissertations but did not include them, as they provided too few details. A total of 2,702 abstracts met the key terms; 2,695 were excluded in first-level screening. Full reports were obtained for the 7 abstracts that met the Phase 1 selection criteria.

At this point we convened an expert consultation to provide feedback on best methods for producing population estimates of PWID for short-term use (i.e., based on available data) and for longer-term use, which may require primary data collection. The group of 10 experts represented persons who directed some of the 7 data systems identified in the Phase 1 automated search, those who had calculated PWID population estimates, and researchers studying PWID populations. For the short term, the expert consultants endorsed the use of meta-analysis of national surveys for making PWID population estimates, acknowledging the limitations of these surveys. National household surveys have the advantage of robust sampling methods and allow for population-based inference; however, they are less robust for measurement of injection drug use due to exclusions (people who are homeless or institutionalized), stigma and social desirability influences related to reporting injection drug use behavior, and small sample sizes of PWID. The expert consultants recommended that we assess the meta-analysis findings against other data sources that may not be included in the meta-analysis.

**Figure 1:**
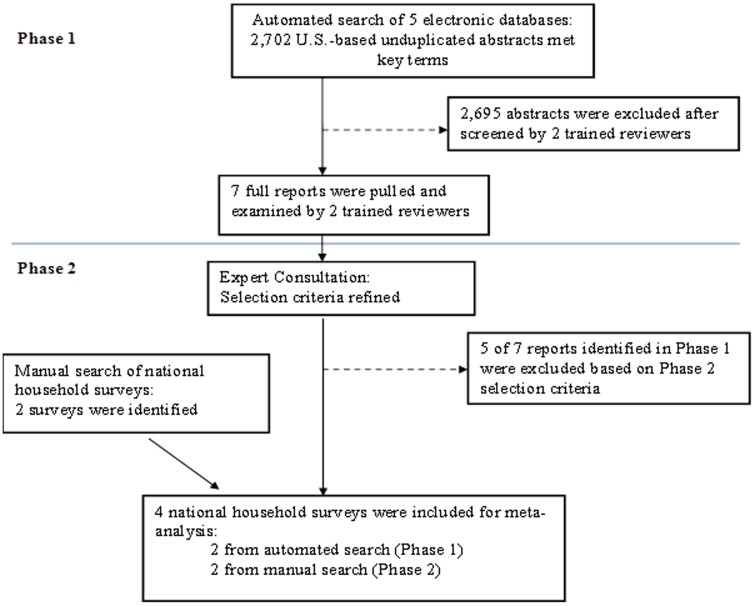
Survey Selection Flowchart for Meta-Analysis.

For the second phase of study selection (Phase 2), we incorporated input from the expert consultants and further restricted our criteria. Studies were included if they were published from 2000 through 2008 to reflect more recent data. Studies were excluded if they provided inadequate description of methods or were not ongoing national, population-based surveys. We restricted to ongoing surveys to allow for multiple years of data to provide a more robust estimate of PWID. In addition, we manually searched for national household surveys that measured injection drug use and applied the Phase 2 selection criteria. Five reports identified in Phase 1 were excluded and 2 national surveys were included from the manual search for a total of 4 surveys used in meta-analysis (Figure 1).

These 4 surveys were the National Survey of Family Growth (NSFG, 2002 and 2006–2008), the National Survey of Drug Use and Health (NSDUH, 2002–2009), the National Health and Nutrition Examination Survey (NHANES, 1999–2008), and the General Social Survey (GSS, 2000–2008). Relevant characteristics of each survey*—*including website addresses for further information about the sampling methods, human subjects review, response rates, and weighting*—*are presented in Table 1. Notably, all surveys were household-based probability samples and 3 of the 4 surveys used audio computer-assisted self-interview to collect information on injection drug use.

**Table 1:** Description of eligible studies for meta-analysis of the population proportion of PWID in the United States.

Study Name	Population Surveyed	Sampling Method	Data used in meta-analysis	Data collection method	Recall Period	Question wording to define PWID
General Social Survey (GSS)	Persons aged ≥18 years, who spoke English or Spanish*	Probability sample	2000, 2002, 2004, 2006, and 2008§	(2000) Face-to-face interviews using paper instruments; (2002–2008) CAPI.	Ever	Have you ever, even once, taken any drugs by injection with a needle (like heroin, cocaine, amphetamines, or steroids)? Do not include anything you took under a doctor's orders.
National Health and Nutrition Examination Survey (NHANES)	Persons aged 12 to 69 years, who spoke English or Spanish†	Stratified, multistage probability cluster design	1999–2008	ACASI	Ever, Past year	**Ever** (1999–2004): Have you ever used a needle to take street drugs? (2005–2008): Have you ever, even once, used a needle to inject a drug not prescribed by a doctor? **Past year**: (1999–2004) In the past 12 months, how many days have you used a needle to take street drugs? (Days greater than 0 indicated injection drug behavior); (2005–2008): How long ago has it been since you last used a needle to inject a drug not prescribed by a doctor? (Time periods of 12 months or less indicated injection drug behavior)
National Survey of Family Growth (NSFG)	Persons aged 15–44 years who spoke English or Spanish	Multistage area probability sample	2002, 2006–2008¶	ACASI	Past year	2002: During the last 12 months, how often have you taken nonprescription drugs using a needle, that is, you took them only for the experience or feeling it caused? This includes ‘shooting up’ and ‘skin-popping.’ (Frequencies greater than 'never' indicated injection drug behavior) 2006: During the last 12 months, how often have you shot up or injected drugs other than those prescribed for you? By shooting up, we mean anytime you might have used drugs with a needle, by mainlining, skin-popping, or muscling. (Frequencies greater than 'never' indicated injection drug behavior)
National Survey of Drug Use and Health (NSDUH)	Persons aged ≥12 who spoke English or Spanish	Stratified, multistage probability cluster sample	2002–2009	ACASI	Ever, Past year	**Ever**: Have you ever, even once, used a needle to inject heroin? **Past year**: How long has it been since you last used a needle to inject heroin? (also asked for a) cocaine, b) methamphetamine, c) other stimulants, and d) other drugs not prescribed/took for the feeling or experience. (1) Within the past 30 days; (2) More than 30 days ago but within past 12 months; (3) More than 12 months ago

CAPI  =  Computer Assisted Personal Interview; ACASI  =  Audio Computer-Assisted Self Interview.

*Spanish GSS interviews began in 2006.

† The public dataset used in this analysis is limited to persons aged 20–69 years, and only those 20–59 answered drug use questions.

§ GSS data are collected every two years.

¶ Between 1973 and 2002, NSFG data were collected in cycles. Since 2006, NSFG data are continuously collected. This analysis included data from Cycle 6 (2002) and 2006 through 2008.

For each survey we analyzed publicly available data using SUDAAN software version 9.1 (RTI International, Research Triangle Park, NC) [13] to account for the complex sample designs. These surveys allowed for 2 estimates of the population proportion of PWID based on recall period: an estimate for “lifetime” injection drug use and an estimate for “past year.” Due to the small number of PWID in each survey year, we aggregated data across years for more robust estimates, resulting in a single estimate for each data source. Prior to aggregating, we examined data for each year and determined no pattern of increasing or decreasing trend during the 2000–2008 time period. Small sample sizes and limited data points (surveys collected data every 2 years or aggregated data into 2-year periods) may have limited power to detect minor changes over time. However, the lack of trend in the 4 surveys is consistent with stable estimates of PWID reported by Tempalski and colleagues for the period 1992–2007 [10]. For the lifetime estimate, NSDUH, NHANES, and GSS contributed data; for the past year estimate, NSDUH, NHANES, and NSFG contributed data.

### Meta-analysis for Estimating the Proportion of PWID in the U.S

To combine the distinct estimates into a summary measure, we applied meta-analytic methods recently extended to survey data [14]. For each recall period (ever, past year) we first multiplied each survey estimate by a weight inversely proportionate to its variance, summed the weighted estimates across studies, and then divided by the sum of the weights.

The studies included for the meta-analysis were sufficiently homogeneous in terms of sampling methods, participants, and outcomes to provide a meaningful summary measure. All were national probability surveys designed to make inference to the U.S. household-based population, and collected self-reported data on injection drug use. Despite these similarities, it is possible that differences in characteristics of the surveys, such as question wording, could result in heterogeneity. We selected random effects models for our analyses because the models assume the studies are a random sample [15], a type of inference that fits the purpose of our study. In our analysis, the measures of injection drug use are not identical across surveys but rather have a distribution; the summary estimate describes the average of the measures and the confidence interval provides an indication of the spread of the distribution of population proportion estimates of PWID. The meta-analysis method developed by Rao et al [14] adds a between-studies variance term in deriving an overall estimate. Heterogeneity of estimates across surveys is indicated with the Q statistic [14] and Higgins' I^2^ index [16]. The Q statistic follows a chi-squared distribution and assesses whether observed differences in results are compatible with chance alone. I^2^ describes the percentage of the variability in effect estimates that is due to heterogeneity rather than sampling error [17]. Values of the Q statistic indicated that the between-studies variance term was a statistically significant source of variability suggesting that the effects being estimated in the different surveys were not identical. We also conducted stratified analyses to further address sources of heterogeneity across surveys.

Stratified analyses for the lifetime estimate were conducted by estimating the population proportion of PWID by sex, race/ethnicity (non-Hispanic white, non-Hispanic black, Hispanic, and other), age group (18–24 years, 25–34 years, 35–49 years, 50–64 years), and for males and females separately by race/ethnic group and by age group. The age groups were determined by the structure of the public use NSDUH data; we restricted all datasets to include only persons aged 18–64 years for comparability. Stratified estimates were for the ever recall period only as there were too few subjects in many of the cells of the cross-classification to produce stable past-year estimates.

We carried out all estimates per Rao's method using Microsoft Excel (2007) and verified them using SAS Version 9.2 (SAS Institute, Cary, NC). We used the Comprehensive Meta-Analysis software version 2 (Biostat, Englewood, NJ) [18], which incorporates assumptions appropriate for synthesizing results from observational studies and clinical trials, to verify results and assess comparable patterns in the data. The estimates from these methods yielded identical results (data not shown).

### Method for Estimating the Numbers of Lifetime PWID in the U.S

We multiplied our newly derived lifetime estimates of the population proportion of PWID by the population estimate from the Census Bureau for persons aged 13 years or older for the 50 states and District of Columbia [19] to obtain an estimated number of PWID.

The population proportions of PWID in the age group 18–24 years were applied to the population aged 13–24 years and the population proportions of PWID in the age group 50–64 years were applied to the population aged 50 years or older. Because smaller percentages of persons in both age groups (13–17 and 65 years or older) are less likely to have ever injected drugs than those aged 18–24 years and 50–64 years, respectively [20], this may result in an over-estimate of the number of PWID and thus an under-estimate of the rates of HIV infection.

### Method for Calculating HIV Disease Rates and Rate Ratios among PWID in the U.S

We calculated HIV rates by dividing the estimated number of cases among PWID (numerator) by the estimated number of PWID (denominator). We calculated two types of HIV rates for the U.S.: 1) diagnosis rates and 2) the rates of PWID living with diagnosed HIV infection. Data sources for rate calculations are shown in Table S1.

For the numerators, we used HIV case surveillance data from all 50 states and the District of Columbia reported to CDC as of June 2012 for adults and adolescents (age 13 years or older at diagnosis) diagnosed with HIV infection in 2011 and for those living with diagnosed HIV infection as of December 2010. The most recently available data were used for both rates; data on persons living with diagnosed HIV infection lags one year behind diagnosis data due to time needed to ascertain deaths [5].

For the denominators, we used the estimated number of lifetime PWID. Denominators were calculated by multiplying census data by the lifetime PWID population proportion derived from the meta-analysis. Our choice to use the estimate based on lifetime injection drug use behavior for calculating rates best corresponds to the HIV surveillance definition, which is injection drug use since 1977 [5]. We used 2011 and 2010 census data, respectively, to determine the number of PWID for the HIV diagnosis rates and rates of living with diagnosed HIV infection (Table S1).

We calculated rate ratios to directly compare rates by sex, race/ethnicity, and age. Females, whites, and the youngest age group (13–24 years) served as the reference groups, respectively.

### Method for Calculating HIV Disease Rate among PWID in Puerto Rico

None of the surveys in the meta-analysis included Puerto Rico in their sampling frames. Because Puerto Rico has a considerable number of HIV diagnoses reported each year and approximately 40% are among PWID [21], we derived a method to calculate HIV rates among PWID in Puerto Rico. A report by Perez and colleagues [22] from a household survey conducted during 2005–2008 indicated that 1.5% of adults aged 21–64 years in Puerto Rico had ever injected drugs (no standard error reported). To calculate a rate, we used the 1.5% population proportion of PWID in Puerto Rico, Puerto Rico population totals from census data [16], and the estimated number of PWID living with diagnosed HIV infection in Puerto Rico in 2010 from CDC's HIV case surveillance data [5]. (Table S1).

### Method for Calculating HCV Disease Rate among PWID in the U.S

For national estimates of the prevalence of HCV infection, CDC relies on data from NHANES, which includes both an interview and HCV antibody testing of the respondent, allowing for calculation of disease rates by identification of persons reporting lifetime injection drug use among those who are anti-HCV positive. We analyzed NHANES data from 2003–2010 for persons aged 40–65 years. This age range captures a majority of those who may have reported injection drug use and roughly corresponds to the age group for which CDC recommends one-time HCV testing without prior ascertainment of HCV infection risk [11]. To calculate the denominator for the rate, the estimate for the population proportion of PWID in the age group 50–64 years was applied to 2011 census data for persons aged 40–65 years (Table S1).

## Results

### Proportion of the Population and Number Estimated to be PWID in the U.S

Table 2 shows the estimated population proportion of lifetime PWID overall and for males and females for each population-based survey and the combined estimates from the meta-analysis. The overall combined estimate for the ever recall period was 2.6% (confidence interval [CI]: 1.8%–3.3%). As noted, Q statistics and I^2^ indicated heterogeneity of results across the surveys (Q_2_ = 45.1, P <.0001, I^2^ = 95.6). The combined estimate for males was 3.6% (CI: 2.4%–4.8%) and for females was 1.6% (95% CI: 1.1%–2.0%). Applying these proportions to the U.S. population age 13 years or older for 2011, we estimate that approximately 6,612,488 adults and adolescents ever injected drugs, with a range from 4,583,188 to 8,641,788 persons; using the sex-specific proportions an estimated 4,532,348 males (range: 3,040,447–6,024,250) and 2,059,709 females (range: 1,513,969–2,605,450) ever injected drugs.

**Table 2:** Estimated proportion of persons who injected drugs (PWID) in their lifetime, by survey and combined by meta-analysis, United States.

Population	Survey	% PWID	95% Confidence Interval	
Males				
	NSDUH	2.7	2.6	2.9
	NHANES	3.3	2.7	3.9
	GSS	4.9	4.2	5.6
	**Combined estimate**	**3.6**	**2.4**	**4.8**
Females				
	NSDUH	1.3	1.2	1.4
	NHANES	1.4	1.1	1.8
	GSS	2.1	1.7	2.5
	**Combined estimate**	**1.6**	**1.1**	**2.0**
**Total**				
	NSDUH	2.0	1.9	2.1
	NHANES	2.3	2.0	2.7
	GSS	3.4	3.0	3.8
	**Combined estimate** *	**2.6**	**1.8**	**3.3**

*Q_2_ = 45.1, p<0.0001, I^2^ = 95.6.

NSDUH  =  National Survey of Drug Use and Health (2002–2009); NHANES = National Health and Nutrition Examination Survey (1999–2008); GSS = General Social Survey (2000 – 2008).

The overall combined estimate for the past year recall period was 0.30% (CI: 0.19%–0.41%) (Table 3). Q statistics and I^2^ indicate heterogeneity of results across these surveys (Q_2_ = 11.7, P <.01, I^2^ = 82.9). The combined past-year estimate for males was 0.36% (CI: 0.26%–0.47%) and for females was 0.21% (CI: 0.10%–0.32%). Applying these proportions to the U.S. population age 13 years or older for 2011, we estimate that approximately 774,434 adults and adolescents (range: 494,605–1,054,263) injected drugs in the past year in the United States.

**Table 3:** Estimated proportion of persons who injected drugs (PWID) in the past year, by survey and combined by meta-analysis, United States.

Population	Survey	% PWID	95% Confidence Interval	
Males				
	NHANES	0.42	0.26	0.66
	NSDUH	0.30	0.26	0.35
	NSFG	0.45	0.29	0.60
	**Combined estimate**	**0.36**	**0.26**	**0.47**
Females				
	NHANES	0.26	0.15	0.46
	NSDUH	0.14	0.12	0.16
	NSFG	0.28	0.16	0.40
	**Combined estimate**	**0.21**	**0.10**	**0.32**
Total				
	NHANES	0.34	0.24	0.48
	NSDUH	0.22	0.19	0.25
	NSFG	0.36	0.27	0.46
	**Combined estimate** *	**0.30**	**0.19**	**0.41**

*Q_2_ = 11.7, p<0.01, I^2^ = 82.9.

NSDUH  =  National Survey of Drug Use and Health (2002–2009); NHANES = National Health and Nutrition Examination Survey (1999–2008); NSFG = National Survey of Family Growth (2002 and 2006–2008).

We calculated lifetime population proportion estimates for male and female PWID by race/ethnicity and by age group (Table 4). The population proportion of PWID was highest among white males (3.8% [CI: 2.7%–4.9%]) and lowest among Hispanic/Latino females (0.7%, [CI: 0.5%–1.0%]). The population proportion of PWID increased with age among those aged 18–49 years.

**Table 4:** Estimated proportion of persons who inject drugs (PWID) in the United States, by sex, race/ethnicity, and age group—meta-analysis of 3 national surveys.*

	Male			Female			Total		
	% PWID	95% CI		% PWID	95% CI		% PWID	95% CI	
**Race/Ethnicity**									
Black/African American	3.4	2.1	4.7	1.5	0.5	2.5	2.5	1.3	3.7
White	3.8	2.7	4.9	1.6	1.4	1.8	2.7	2.1	3.3
Hispanic/Latino	2.3	1.4	3.2	0.7	0.5	1.0	1.6	1.0	2.2
Other	2.3	0.8	3.8	1.3	0.9	1.7	1.7	1.0	2.4
**Age Group (years)**									
18–24	1.8	1.0	2.6	1.0	0.8	1.1	1.3	0.9	1.6
25–34	3.4	1.8	5.1	1.2	1.0	1.4	2.4	1.4	3.4
35–49	4.1	3.0	5.2	2.0	1.4	2.5	3.0	2.2	3.9
50–64	3.5	2.7	4.4	1.2	0.8	1.6	2.5	1.7	3.3
**Total**	**3.6**	**2.4**	**4.8**	**1.6**	**1.1**	**2.0**	**2.6**	**1.8**	**3.3**

CI = Confidence Interval.

* Surveys that measured lifetime injection drug use used in the meta-analysis: National Survey on Drug Use and Health (NSDUH), National Health and Nutrition Examination Survey (NHANES), and General Social Survey (GSS).

### HIV Disease Rates and Rate Ratios among PWID in the U.S

Rates of diagnosis of HIV infection among PWID and rates of PWID living with a diagnosis of HIV infection are presented in Tables 5 and 6. The rate of diagnosis of HIV infection for PWID was 55 per 100,000 PWID (CI: 42–80); the rate of PWID living with a diagnosis of HIV infection was 2,147 per 100,000 PWID (CI: 1,643–3,098). The rates for males were lower than those for females for both measures, but not significantly different.

**Table 5:** Diagnoses of HIV infection among persons who inject drugs (PWID), by sex- - United States, 2011.

	No. of PWID cases*	Rate**	95% CI	Rate Ratio	95% CI
Males	2,220	49	37–73	0.7	0.4–1.3
Females	1,428	69	55–94	1.0	—
Total	3,648	55	42–80		

Note. Data include persons age 13 years and older with a diagnosis of HIV infection regardless of stage of disease at diagnosis. CI = confidence interval.

* Number of cases diagnosed with HIV infection or living with HIV infection was statistically adjusted to account for reporting delays and missing risk factor information, but not for incomplete reporting.

**Per 100,000 PWID.

**Table 6:** Persons who inject drugs (PWID) living with HIV, by sex - United States, 2010.

	No. of PWID cases*	Rate**	95% CI	Rate Ratio	95% CI
Males	86,515	1927	1450–2873	0.7	0.4–1.4
Females	54,214	2653	2098–3610	1.0	—
Total	140,729	2147	1643–3098		

Note. Data include persons age 13 years and older with a diagnosis of HIV infection regardless of stage of disease at diagnosis.

* Number of cases diagnosed with HIV infection or living with HIV infection was statistically adjusted to account for reporting delays and missing risk factor information, but not for incomplete reporting.

**Per 100,000 PWID.

The rate ratios illustrate disparities by race/ethnicity. Comparing black male PWID to white male PWID, the estimated rate of diagnoses of HIV infection was 7–29 times as high (Table S2) and the estimated rate of living with a diagnosis of HIV infection was 9–38 times as high (Table S3); comparing Hispanic/Latino to white male PWID, these rates were 4–17 and 5–20 times as high, respectively (Tables S2 and S3). Comparing black female PWID to white female PWID, the estimated rate of diagnosis of HIV infection was 5–29 times as high (Table S2) and the estimated rate of living with a diagnosis of HIV infection was 6–42 times as high (Table S3); comparing Hispanic/Latino to white female PWID, these rates were 3–8 and 4–12 times as high, respectively (Tables S2 and S3). By age, the rate of PWID living with a diagnosis of HIV was higher among older age groups than those 13–24 years for males and for females (Table S2).

### HIV Infection Rate among PWID in Puerto Rico

The method we used to estimate the rate of persons living with a diagnosis of HIV infection among PWID in Puerto Rico in 2010 produced a rate of 14,494 per 100,000 PWID (or a prevalence of 14.5%).

### HCV Infection Rate among PWID in the U.S

The prevalence rate of HCV infection among PWID aged 40–65 years was 43,126 per 100,000 PWID (CI: 34,024–58,875).

## Discussion

Using data from national population-based U.S. surveys, we estimated that persons who ever injected drugs comprised 2.6% (CI: 1.8%–3.3%) of the U.S. population. This represents approximately 6,612,488 million PWID (range: 4,583,188–8,641,788) aged 13 years or older in 2011. Although PWID comprise 3% or less of the U.S. population, they account for 22% of all persons living with HIV infection [2]. Our estimates also quantified the recognized disparity of HIV disease rates among black and Hispanic/Latino male and female PWID when compared with white male and female PWID.

The rates we calculated for living with a diagnosis of HIV infection in 2010 represent approximately 2% among male PWID and 3% among female PWID. In recent years, national HIV seroprevalence data among PWID have originated primarily from NHANES [23], which was a data source in our meta-analysis. More recent data on HIV seroprevalence among past-year PWID in 20 cities with high AIDS prevalence was 9%, with similar patterns of higher prevalence among black and Hispanic/Latino compared to white PWID [24]. However, those data on past-year PWID are not directly comparable to our rates which were based on lifetime PWID.

Our past year estimate represents about 774,434 PWID in 2011 (range: 494,605–1,054,263). Tempalski and colleagues found a 2007 population estimate for PWID of approximately 1,500,000 with minimum and maximum estimates of approximately 1,300,000 and 1,700,000 million [10]. In view of the differences between our study and that by Tempalski and colleagues in terms of methods (meta-analysis vs. multiplier methods), datasets (national surveys vs. drug and HIV testing data), and time periods (2000–2008 vs. 1992–2007), and the lack of an accepted gold standard method for PWID population size estimates [6], it is unclear whether our estimate represents under-estimation or the Tempalski method represents over-estimation. The use of any PWID population size estimate should be accompanied by acknowledgement of the limitations of the methods and data sources.

Our rate of adults and adolescents living with a diagnosis of HIV infection among PWID in Puerto Rico (14%) is higher than that published by Perez and colleagues (2.8% [CI 0.6%–12.4%]) [22]. The estimate of the population proportion of PWID in Puerto Rico (1.5%), based on a single household survey, could be an under-estimate. In addition, the investigators cautioned that the small number of HIV-infected persons limited their ability to make reliable prevalence estimates stratified by injection drug use [22].

Our HCV infection prevalence rate among PWID aged 40–65 years was 43,126 per 100,000 population, reflecting the substantial impact of injection drug use on acquiring HCV infection. The HCV infection prevalence we found (43.1%) was similar to a previous NHANES estimate of 48% [4]; the higher NHANES prevalence may be due to the inclusion of PWID in a broader age range than our estimate. The prevalence estimate points to the importance of national efforts to raise awareness of HCV testing among persons who have injected drugs [25]. In an era of improved treatment, it is also important that those who are infected are linked to appropriate care [11]. CDC recommends integrated prevention services for PWID, which address risk for HIV and HCV infections and are expected to result in increased access to services, improved timeliness of service delivery, and increased effectiveness of prevention efforts [26].

Our results are subject to several limitations. While the study designs are robust in the 4 national surveys, they are hampered by small proportion of participants reporting injection drug use. Because PWID are a small proportion of the general population, obtaining adequate numbers to produce stable estimates is difficult without very large sample sizes. This difficulty is exacerbated when estimates are stratified by sex, age, or race/ethnicity. In addition, the illicit nature of and stigma associated with injection drug use may have resulted in under-reporting of this behavior; however this bias should be mitigated in part by use of ACASI for most surveys included in our analysis. A second limitation is coverage bias. The surveys in the meta-analysis exclude individuals without stable housing. Given that a high proportion of PWID are unstably housed [27], [28], they are likely underrepresented in our analysis. This coverage bias would result in an under-estimate of the population proportion of PWID and an over-estimate of disease rates. A third limitation is the degree of heterogeneity among surveys. Although all surveys are population-based, the sampling methods, age range, and question wording vary across surveys. We used random-effects models to account for variance beyond sampling errors. As noted, the rates among those aged 13–24 years and 50 years or older may be under-estimates given that the meta-analysis was limited to those aged 18–64 years. Other limitations are inherent from the surveillance data used in the rate calculations [5]. The HCV infection data are subject to the limitations of NHANES [4].

Given the potential factors affecting the data in the 4 surveys and the surveillance data, the population estimate and disease rates should be presented with acknowledgement of their limitations and interpreted in the context of the confidence intervals presented; wider confidence intervals for some groups indicate less precision in the estimates. The estimation method presented here (meta-analysis results of ongoing, national survey data) represents one method, as our expert consultants recognized, for estimating the size of the PWID population in the United States. As more research is conducted to estimate population size of groups at risk for HIV and HCV infection, we will consider using different methods in the future, should they prove more accurate or more tractable than meta-analysis of national survey data. These methods include capture-recapture, using data collected from the population at risk, and network scale-up method based on data collected from the general population [6]. For purposes of determining whether there are better methods or data sources for use in the future, our expert consultants recommended small-scale feasibility studies of multiple estimation methods in one geographic area to be able to compare estimates generated by these different methods (e.g., national survey, network scale-up, and capture-recapture). However, for the short term, synthesizing national surveys was recommended by the expert consultants, the approach we took in this paper.

Estimating the population proportion of PWID allowed calculation of rates of HIV and HCV infection, which quantifies the disproportionate impact among PWID nationally. Trends from population-based surveys will be monitored as part of CDC's behavioral surveillance analyses and the meta-analysis can be updated as new data emerge. For HIV infection, rates can be calculated on an annual basis with the most recent surveillance data. Other disease metrics can be used to calculate rates, such as HIV incidence [1] or national HIV prevalence estimates [2], which include persons with undiagnosed HIV infection. Because we calculated past year as well as lifetime estimates, others can use either, depending on which best fits their needs. However, our estimates may not be well suited for calculating disease rates at the state or local level as the population sizes of PWID vary across the U.S. [10].

The best available data must be used to guide decision-making for disease prevention. The estimate of the number of PWID (lifetime and past year) in the U.S. and quantifying the burden of disease and disparities among PWID can be particularly important for planning and evaluating programs serving disproportionately affected populations and addressing health inequities at the national level. The estimate of the number of PWID in the U.S. and resulting rates are important additions to cost effectiveness and other data used to make critical decisions about resources for prevention of HIV and HCV infections.

## Supporting Information

Table S1 **Data sources used for rate calculations.**
(DOCX)

Table S2 **Number, rate, and rate ratio of diagnoses of HIV infection among adult and adolescent persons who inject drugs (PWID), by selected characteristics—United States, 2011.**
(XLSX)

Table S3 **Number, rate, and rate ratio of adult and adolescent persons who inject drugs (PWID) living with diagnosed HIV infection, by selected characteristics—United States, 2010.**
(XLSX)

Checklist S1 **PRISMA Checklist.**
(DOC)
