# Supplementary material for: Estimating the Number of Persons Who Inject Drugs in the United States by Meta-Analysis to Calculate National Rates of HIV and Hepatitis C Virus Infections
Source: PLoS One. 2014 May 19;9(5):e97596. doi: 10.1371/journal.pone.0097596 (PMC4026524; doi:10.1371/journal.pone.0097596)
Supplement: Table S1 — Data sources used for rate calculations. (DOCX) [file pone.0097596.s001.docx]

**Table S1. Data sources for rate calculations**

| **Rate Measure** | **Numerator** | **Denominator** | |
| --- | --- | --- | --- |
|  |  | **PWID population proportion** | **Census year [16]** |
| HIV diagnoses in the United States | HIV case surveillance [5] | Meta-analysis | 2011 |
| Persons living with diagnosed HIV in the United States | HIV case surveillance [5] | Meta-analysis | 2010 |
| HIV prevalence in Puerto Rico | HIV case surveillance [5] | Puerto Rico household survey [19] | 2010 |
| HCV prevalence in the United States | NHANES* | Meta-analysis | 2010 |

Note: Numerators were obtained directly from the data source indicated. Denominators were calculated as (PWID population proportion * census data) to obtain a number of PWID. Rates were calculated per 100,000 PWID. Calculations for HIV diagnoses, living with diagnosed HIV, and HCV prevalence were based on the most recent year available. HIV prevalence in PR was calculated for 2010 to be consistent in time period for the calculation of living with diagnosed HIV infection for United States.

* NHANES source: www.cdc.gov/nchs/nhanes
